# Mutational analysis of uterine cervical cancer that survived multiple rounds of radiotherapy

**DOI:** 10.18632/oncotarget.25982

**Published:** 2018-08-24

**Authors:** Endang Nuryadi, Yasushi Sasaki, Yoshihiko Hagiwara, Tiara Bunga Mayang Permata, Hiro Sato, Shuichiro Komatsu, Yuya Yoshimoto, Kazutoshi Murata, Ken Ando, Nobuteru Kubo, Noriyuki Okonogi, Yosuke Takakusagi, Akiko Adachi, Mototaro Iwanaga, Keisuke Tsuchida, Tomoaki Tamaki, Shin-ei Noda, Yuka Hirota, Atsushi Shibata, Tatsuya Ohno, Takashi Tokino, Takahiro Oike, Takashi Nakano

**Affiliations:** ^1^ Department of Radiation Oncology, Gunma University Graduate School of Medicine, Gunma, Japan; ^2^ Department of Radiotherapy, Dr. Cipto Mangunkusumo National General Hospital, Jakarta, Indonesia; ^3^ Department of Medical Genome Sciences, Research Institute for Frontier Medicine, Sapporo Medical University, Sapporo, Japan; ^4^ Education and Research Support Center, Gunma University Graduate School of Medicine, Gunma, Japan; ^5^ Gunma University Heavy Ion Medical Center, Gunma, Japan

**Keywords:** uterine cervical cancer, radioresistance, next-generation sequencing, KRAS, SMAD4

## Abstract

Radiotherapy is an essential component of cancer therapy. Despite advances in cancer genomics, the mutation signatures of radioresistant tumors have not yet been fully elucidated. To address this issue, we analyzed a unique set of clinical specimens from a uterine cervical cancer that repeatedly locally recurred after multiple rounds of radiotherapy. Exon sequencing of 409 cancer-related genes in the treatment-naïve tumor and the tumors that recurred after initial and secondary radiotherapy identified (i) activating mutations in *PIK3CA* and *KRAS*, and putative inactivating mutations in *SMAD4*, as trunk mutation signatures that persisted over the clinical course; and (ii) mutations in *KMT2A*, *TET1*, and *NLRP1* as acquired mutation signatures observed only in recurrent tumors after radiotherapy. Comprehensive mining of published *in vitro* genomics data pertaining to radiosensitivity revealed that simultaneous mutations in *KRAS* and *SMAD4*, which have not been described previously in uterine cervical cancer, are associated with cancer cell radioresistance. The association between this mutation signature and radioresistance was validated by isogenic cell-based experiments. These results provide proof-of-principle for the analytical pipeline employed in this study, which explores clinically relevant mutation signatures for radioresistance, and demonstrate that this approach is worth pursuing with larger cohorts in the future.

## INTRODUCTION

Radiotherapy is an essential component of cancer therapy [1, 2]. In the field of radiotherapy, clinical management aimed at improving treatment precision has generally focused on delivering radiations to a defined tumor target [3]. On the other hand, in the biological context, the factors used clinically for optimization of treatment planning include tumor size, imaging features, and histopathological typing [3, 4]. The response of tumors to radiotherapy varies even among tumors for which these factors are similar, highlighting the need for additional indices to improve prediction of tumor radiosensitivity.

Precision cancer medicine, which uses the genetic information of individual tumors to guide treatment, is currently emerging in the field of clinical oncology [5]. Advances in next-generation sequencing technologies have enabled the identification of multiple genetic alterations that make tumor cells responsive to molecularly targeted drugs [6–9]. However, the mutation signatures of tumors resistant to radiotherapy have not been fully elucidated. Such mutation signatures would contribute to facilitation of precision radiotherapy, enabling clinicians to stratify patients with radioresistant tumors into treatments with high-intensity radiation modalities, e.g., carbon ion radiotherapy [4].

To address this issue, we investigated the mutation signatures of a unique set of clinical specimens from a uterine cervical cancer that repeatedly locally recurred after multiple rounds of radiotherapy. Exon sequencing of 409 cancer-related genes in treatment-naïve and multiply-recurrent tumors revealed rare simultaneous mutations in *KRAS* and *SMAD4*. In addition, we validated the association between this mutation signature and radioresistance by performing meta-analysis of published *in vitro* data and isogenic cell-based experiments.

## RESULTS

### Mutation profiles of radioresistant tumors

To investigate mutation signatures associated with radioresistance, we analyzed a series of tumors collected from a patient with uterine cervical cancer who experienced repeated local recurrence after multiple rounds of curative radiotherapy (Table 1). Given that the 5-year local control rate for uterine cervical cancer treated with curative radiotherapy is as high as >90%, this case is considered extremely radioresistant [10]. At the time of diagnosis, the patient was 34 years old and the FIGO stage was IB2. The tumor was positive for HPV-16. The pathological diagnosis (according to the UICC pTNM, 6th edition) was a mucinous adenocarcinoma of the uterine cervix, intestinal plus endocervical type, pT1b2N1MX, G2 (R0, Stage IIIB). Surgery comprised radical hysterectomy and bilateral salpingo-oophorectomy. Lymphatic invasion, vascular invasion, and metastatic foci were observed in the right ovary. Adjuvant chemotherapy comprised six courses of mitomycin C (10 mg/body on Day 1), etoposide (80 mg/body on Days 1, 3, and 5), and cisplatin (45 mg/body on Day 1). Radiotherapy for the first recurrence comprised external body radiotherapy (50 Gy in 25 fractions delivered to the whole pelvis, using the center-seal technique for the latter 20 Gy) and intracavitary brachytherapy (23 Gy delivered in 4 fractions). Radiotherapy for the second recurrence comprised interstitial brachytherapy (30 Gy delivered in 5 fractions). Radiotherapy for the third recurrence comprised interstitial brachytherapy (30 Gy delivered in 5 fractions).

**Table 1:** Clinical course of the patient and timing of sample collection

Event	Sample	Treatment	Months
Diagnosis	T1		0
	Normal	Surgery	1
		Adjuvant chemotherapy	3
Recurrence at vaginal stump			11
		Radiotherapy (EBRT+ICBT)	13
Recurrence at vaginal stump	T2		31
		Radiotherapy (ISBT)	32
Recurrence at vaginal stump	T3		46
		Radiotherapy (ISBT)	47
Deceased			71

Abbreviations: EBRT, external body radiotherapy; ICBT, intracavitary brachytherapy; ISBT, interstitial brachytherapy. Treatment details are described in Supplementary Text.

Using the Ion AmpliSeq Comprehensive Cancer Panel (Thermo Fisher Scientific), we performed semiconductor-based next-generation sequencing of exons of 409 cancer-related genes in the treatment-naïve tumor (T1), recurrent tumor after initial radiotherapy (T2), recurrent tumor after secondary radioherapy (T3), and normal tissue of the uterine cervix (as a Control) (Table 1 and Supplementary Figure 1). T2 and T3 were diagnosed as local recurrence based on the fact that pathological findings for T2 and T3 were consistent with T1.

After quality filtering, the number of sequencing reads per sample was 10.6 million (Supplementary Table 1). Average coverage depth was 616 reads per base (Supplementary Table 1). Somatic mutations and copy number variations (CNVs) were determined using the analytical workflow described in *Identification of somatic mutations and CNVs* in MATERIALS AND METHODS. In total, we identified 91 somatic non-synonymous mutations and 394 CNV regions.

**Figure 1:**
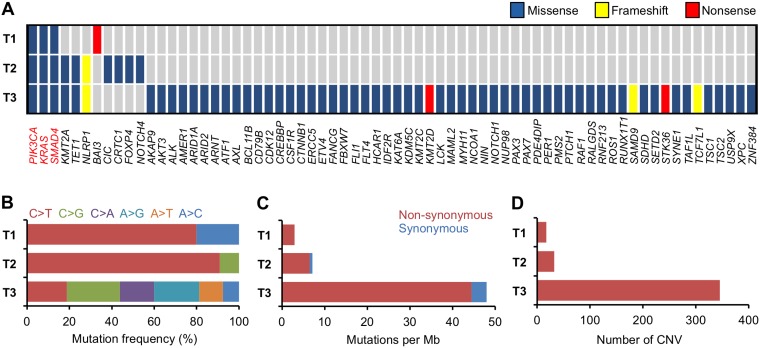
Summary of somatic alterations in treatment-naïve and recurrent tumors (**A**) Mutated genes, according to mutation type. Genes found to be mutated in T1−T3 are indicated in red. (**B**) Mutation spectrum of single-nucleotide substitutions. (**C**) Number of mutations. (**D**) Number of CNV regions.

**Figure 2:**
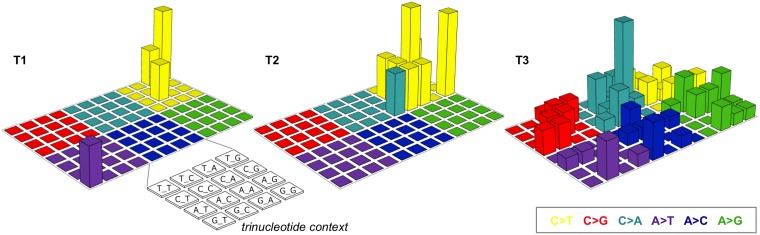
Lego plots of mutational patterns in a three-base context The somatic single-nucleotide variants identified in T1–3 are categorized into six groups based on the base substitution type and are shown in different colors. Each group is further classified into 16 subgroups based on the 5’ and 3’ neighboring bases [12, 14].

T1 harbored the *PIK3CA* E545K mutation (Figure 1A and Supplementary Table 2). *PIK3CA* is the gene most frequently mutated in uterine cervical cancer (14−26% of cases), and E545K is one of three hotspots for activating mutations in this gene [11−13]. T1 also had a high frequency (i.e., 60%; 3/5) of single-nucleotide substitutions in cytosines preceded by thymines, a characteristic of APOBEC mutagenesis, which is the predominant source of mutations in uterine cervical cancer (Figures 1B and 2) [11, 12, 14]. These observations suggested that the genetic characteristics of the treatment-naïve tumor were typical of uterine cervical cancer.

Interestingly, T1−T3 had mutations in *KRAS* and *SMAD4* in common (Figure 1A and Supplementary Table 2). *KRAS* G12D is a well-studied oncogenic driver, whereas the co-occurrence of R361C and R361H in *SMAD4* indicated biallelic inactivation of this tumor suppressor [15−18]. The persistence of these mutations in T1−T3 indicates that they played pivotal roles in the radioresistance that enabled survival of the tumor through repeated rounds of radiotherapy.

Mutations in *KMT2A*, *TET1*, and *NLRP1* were present in both T2 and T3, but not in T1 (Figure 1A and Supplementary Table 2). In addition, amplification of *PIK3CA* and *FGFR2* genes was observed in both T2 and T3, but not in T1. These observations suggest that the gene alterations are generated, or accumulated, as the tumor survives through the initial round of radiotherapy. Therefore, these gene alterations may be involved in acquisition of radioresistance.

The number of somatic mutations and CNVs in T1 and T2 was comparable, but was higher in T3 than in T2 (Figure 1A, 1C, 1D and Supplementary Table 1). Interestingly, T3 exhibited an unbiased spectrum of single-nucleotide substitutions in contrast with T1 and T2 (Figures 1B and 2).

### Association of simultaneous mutations in *KRAS* and *SMAD4* with radioresistance

**Figure 3:**
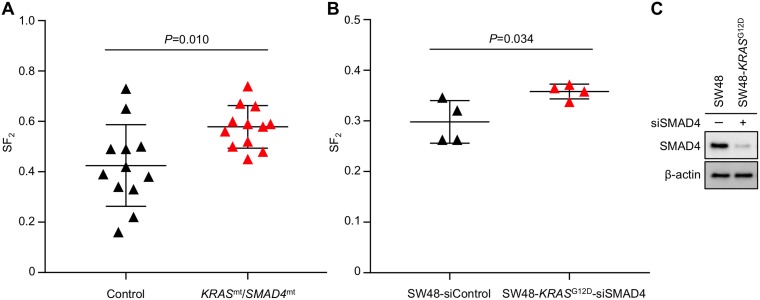
Higher SF_2_ is associated with simultaneous mutations in *KRAS* and *SMAD4* (**A**, **B**) SF_2_ values (assessed in a clonogenic assay) for 12 *KRAS*^mt^*/SMAD4*^mt^ and 12 Control cell lines (obtained from the literature) (A) and those for siControl-treated SW48 and siSMAD4-treated SW48-*KRAS*^G12D^ cells (experiments were performed in quadruplicate) (B). Bars denote the mean ± standard deviation (for details, see *Acquisition of SF_2_ data from the literature* in MATERIALS AND METHODS and Supplementary Table 6). (**C**) Immunoblots showing expression of SMAD4 and β-actin (as a loading Control) in siControl-treated SW48 and siSMAD4-treated SW48-*KRAS*^G12D^ cells.

Among the identified somatic alterations, the simultaneous mutations in *KRAS* and *SMAD4* caught our attention because (i) they have not been reported in uterine cervical cancers, and (ii) their persistence in T1−T3 indicates that this mutation signature contributed to the extreme radioresistance of the tumor. Hence, we investigated the association of this mutation signature with radiosensitivity in published *in vitro* data. Despite an intensive literature search, we were unable to identify uterine cervical cancer cell lines with simultaneous mutations in *KRAS* and *SMAD4*, supporting the idea that this mutation signature is rare among uterine cervical cancers. We then expanded our search to 1039 cell lines derived from various cancers registered in the Cancer Cell Line Encyclopedia (CCLE). This comprehensive search identified 12 cell lines simultaneously carrying (i) gene amplification and/or putative driver mutations in *KRAS* and (ii) homozygous deletion or truncation mutations in *SMAD4* (hereafter referred to as *KRAS*^mt^/*SMAD4*^mt^ cell lines) for which *in vitro* radiosensitivity has been assessed in clonogenic assays (Table 2). As a control, we randomly selected 12 cancer type–matched cell lines without simultaneous mutations in *KRAS* and *SMAD4* (hereafter referred to as Control cell lines) for which published *in vitro* radiosensitivity data were available (Table 2). The prevalence of *KRAS* and *SMAD4* mutations in the Control cell lines was 50% and 17%, respectively, whereas that in cancer type– and sample size–matched clinical tumor populations was 48% and 2.5%, respectively. This indicates that the randomly selected Control cell lines reflect the profiles of clinical tumors in terms of *KRAS* mutations, with a relatively high prevalence of *SMAD4* mutations (for details, see *Identification of KRAS^mt^/SMAD4^mt^ and Control cell lines in CCLE* in MATERIALS AND METHODS) (Table 2 and Supplementary Table 3). The surviving fraction after exposure to 2-Gy irradiation, as assessed in clonogenic assays (SF_2_), is relevant with respect to the tumor response to radiotherapy; the precision of SF_2_ is sufficient for inter-assay comparison [19, 20]. Based on this evidence, we obtained all available SF_2_ data for the *KRAS*^mt^/*SMAD4*^mt^ and Control cell lines by performing a comprehensive literature search (for details, see *Acquisition of SF_2_ data from the literature* in MATERIALS AND METHODS) and compared SF_2_ data between the two groups (Table 2 and Supplementary Table 4). We found that SF_2_ in the 12 *KRAS*^mt^/*SMAD4*^mt^ cell lines was significantly higher than in the 12 Control cell lines (*P* = 0.010) (Figure 3A), indicating that simultaneous mutations in *KRAS* and *SMAD4* are associated with radioresistance.

**Table 2:** *KRAS*^mt^/*SMAD4*^mt^ and Control cell lines

Cell line	Group	Cancer type	*KRAS*	*SMAD4*	SF_2_
AsPC-1	*KRAS*^mt^/*SMAD4*^mt^	pancreatic	G12D	HD	0.66 ± 0.13
PK-1	*KRAS*^mt^/*SMAD4*^mt^	pancreatic	G12D	HD	0.50
SW1990	*KRAS*^mt^/*SMAD4*^mt^	pancreatic	G12D	HD	0.56 ± 0.19
Capan-1	*KRAS*^mt^/*SMAD4*^mt^	pancreatic	G12V	S343*	0.52 ± 0.24
CFPAC-1	*KRAS*^mt^/*SMAD4*^mt^	pancreatic	AMP, G12V	HD	0.58
PSN1	*KRAS*^mt^/*SMAD4*^mt^	pancreatic	AMP, G12R	HD	0.48 ± 0.00
T84	*KRAS*^mt^/*SMAD4*^mt^	colorectal	G12D	HD	0.74 ± 0.28
SW403	*KRAS*^mt^/*SMAD4*^mt^	colorectal	G13V	HD	0.59
SW480	*KRAS*^mt^/*SMAD4*^mt^	colorectal	AMP, G12V	HD	0.67 ± 0.07
SW620	*KRAS*^mt^/*SMAD4*^mt^	colorectal	AMP, G12V	HD	0.59 ± 0.16
AGS	*KRAS*^mt^/*SMAD4*^mt^	stomach	G12D	HD	0.45 ± 0.06
SW1573	*KRAS*^mt^/*SMAD4*^mt^	lung	G12C	HD	0.60 ± 0.08
Capan-2	Control	pancreatic	G12V	WT	0.49 ± 0.04
MIAPaCa-2	Control	pancreatic	G12C	WT	0.49 ± 0.10
PANC-1	Control	pancreatic	AMP	WT	0.73 ± 0.20
HCT 116	Control	colorectal	G13D	WT	0.42 ± 0.10
LoVo	Control	colorectal	G13D	WT	0.38 ± 0.10
LS180	Control	colorectal	G12D	WT	0.50
RKO	Control	colorectal	WT	WT	0.33 ± 0.13
SW48	Control	colorectal	WT	WT	0.22 ± 0.06
NCI-N87	Control	stomach	WT	HD	0.39 ± 0.15
MKN-45	Control	stomach	WT	HD	0.65 ± 0.25
NCI-H1437	Control	lung	WT	WT	0.34
NCI-H1648	Control	lung	WT	WT	0.16

Abbreviations: AMP, amplification; HD, homozygous deletion; WT, wild type. SF_2_ data were obtained from previous publications, and the average ± standard deviation (if available) value for each cell line is shown (for details, see *Acquisition of SF_2_ data from the literature* in MATERIALS AND METHODS).

Finally, we examined the association between the *KRAS*^mt^/*SMAD4*^mt^ signature and radioresistance measured in isogenic cell-based experimental systems. For these experiments, we used *KRAS* G12D-knock-in cells (SW48-*KRAS*^G12D^) treated with SMAD4-siRNA (siSMAD4) which mimics loss-of-function mutations in *SMAD4*, and the parental SW48 cell line (as the Control) (Table 2). We found that the SF_2_ values for siSMAD4-treated SW48-*KRAS*^G12D^ cells were significantly higher than those for SW48 cells (*P* = 0.034) (Figure 3B and 3C). These data support an association between radioresistance and simultaneous mutations in *KRAS* and *SMAD4*.

## DISCUSSION

The profiles of mutations that occur over the clinical course of cancer have been studied extensively in the context of recurrence after chemotherapy. For example, *EGFR* T790M is enriched after treatment with EGFR tyrosine kinase inhibitors, and this mutation contributes to resistance to this type of therapy [21]. Recently, diverse genetic branching was reported in spatiotemporally different tumors in a single patient [22]. From this perspective, the present study is the first to compare the mutation profiles of treatment-naïve tumors with those of multiply-recurrent tumors collected from the same disease site following multiple rounds of radiotherapy. Our results highlighted (i) activating mutations in *PIK3CA* and *KRAS*, and putative inactivating mutations in *SMAD4,* as trunk mutation signatures that persisted over the clinical course; and (ii) mutations in *KMT2A*, *TET1*, and *NLRP1*, and gene amplification of *PIK3CA* and *FGFR2*, as acquired mutation signatures observed only in recurrent tumors after radiotherapy. Comprehensive mining of published *in vitro* data revealed that simultaneous mutations in *KRAS* and *SMAD4* are associated with cancer cell radioresistance. Although this was a pilot study that analyzed only one case, these data provide proof-of-principle for the analytical pipeline that we employed, which explores clinically relevant mutation signatures for radioresistance, indicating that this approach is worth pursuing in larger cohorts in the future.

Several studies have demonstrated predictive biomarkers for radiosensitivity [23]. In many cases, radiosensitivity is predicted based on gene expression profiles of frozen tumor tissues, which are generally difficult to collect in routine clinical practice. Importantly in this regard, the *KRAS^mt^/SMAD4^mt^* signature can be assessed using formalin-fixed paraffin-embedded (FFPE) tissues, which are routinely used for pathological diagnosis, indicating that this approach is suitable for application in the clinic. The predictive value of the *KRAS^mt^/SMAD4^mt^* signature for radioresistance should be verified further in clinical studies.

To date, two large-scale whole-exome sequencing studies of uterine cervical cancer have been conducted to identify its genomic landscape [8, 9]. According to these studies, the genomic profiles of uterine cervical cancer are characterized by recurrent alterations in *PIK3CA*, *EP300*, *FBXW7*, *PTEN*, *HLA-B*, and *KRAS* (in 14−26%, 11−12%, 10−11%, 7-8%, 6-8%, and 3–9% of cases, respectively), along with a predominant APOBEC mutagenesis pattern. Mutations in *PIK3CA* and *KRAS* have been studied using targeted sequencing approaches, which identified mutation frequencies comparable with those observed in whole-exome sequencing studies [24−28]; while alterations in *SMAD4* was identified in only one of the whole-exome sequencing study at 5% of cases [11]. Concomitant alterations in *KRAS* and *SMAD4* have not been reported for uterine cervical cancer; indeed, data combined from different types of tumors suggest that such alterations are rare (i.e., 1.2%; 279/22928) (Supplementary Table 5 and Supplementary Figure 2). Nevertheless, it is noteworthy that pancreatic cancer, which is clinically radioresistant [29], shows the highest frequency of alterations (24%). Interestingly, a previous study demonstrates that treatment of hTERT-immortalized human pancreatic duct cells in which KRAS is overexpressed and SMAD4 is suppressed upregulates NF-kB [30]. Since NF-kB regulates multiple anti-apoptotic genes and is considered to be a major contributor to radioresistance [31], it is possible that simultaneous mutations in *KRAS* and *SMAD4* (as observed in the present study) contributed to the radioresistant nature of the tumor via this mechanism.

KMT2A is a member of the KMT2 complex, which methylates lysine 4 on histone H3 (H3K4) to promote genome accessibility and transcription [32]. A recent study reports broad trimethylation of H3K4 in pan-cancer tumor suppressor genes, including *TP53*, in normal cells, and shortage of the trimethylation pattern in cancer cells in opposite [33]. In cancer, common mutations of *KMT2A* are nonsense, frameshift, and missense mutations; this suggests that *KMT2A* mutations observed in cancer contribute to loss-of-function of KMT2A [32], whereas suppression of *TP53* contributes to resistance of cancer cells to X-rays [34]. Taken together, the evidence suggests that the *KMT2A* mutations observed herein might play roles in acquisition of radioresistance by tumor cells through loss-of-function of KMT2A, which contributes to suppression of *TP53* and other tumor suppressor genes, transcription of which is regulated by methylation on H3K4.

TET1 is an enzyme that catalyzes successive oxidation of 5-methylcytosine to 5-hydroxymethylcytosine, 5-formylcytosine, and 5-carboxylcytosine to promote DNA demethylation [35]. Mutations in *TET1* occur in a wide variety of solid tumors, albeit at relatively low frequency (0.1−10% of cases) [35]. However, we do not have solid evidence on how *TET1* mutations contribute to onset and maintenance of tumors, and it is unclear whether *TET1* mutations confer a selective advantage, or whether they represent inconsequential passenger mutations; this warrants further research [35].

Studies suggest that FGF2 upregulates expression of anti-apoptotic proteins such as BCL-2 by activating S6 kinase within the PI3K-AKT signaling pathway, thereby promoting resistance to chemotherapy [36]. Amplification of *PIK3CA* and *FGFR2* might contribute to acquisition of radioresistance by the tumor in the present study. In addition, amplification of *PIK3CA* and *FGFR2* in T2 and T3, combined with activating mutations in *KRAS* and *PIK3CA* in T1−3, indicates a critical role for the RAS-PI3K axis in tumor survival through multiple rounds of radiotherapy [37].

Several mutations detected in T1 or T2 were not detected in T3. It is possible that radiotherapy induces nucleotide modifications in these genes in some cancer cells, which was followed by clonal selection. Another possibility is that differences in the mutational profiles observed in these samples are based on genetic heterogeneity within the tumor, which was retained over the clinical course. Further studies are warranted.

The present study has the following limitations. First, we used a commercially available gene panel that contains only 409 cancer-related genes. Therefore, we were not able to investigate the mutational status of genes not listed in the panel. It should be noted that a number of mutations in genes not listed in the panel do occur in uterine cervical cancers [38]. Further studies based on whole-exome sequencing will help to identify the mutational signature associated with radioresistance in uterine cervical cancer. Second, we analyzed only one case, due to the rarity of patients who receive multiple rounds of radiotherapy at the same biopsy-accessible disease site. Third, we did not investigate the mechanisms underlying the radioresistance associated with the *KRAS^mt^/SMAD4^mt^* signature; we considered such an effort to be outside the scope of the present study, which primarily aimed to discover clinically applicable mutation signatures associated with radioresistance. Fourth, we diagnosed T2 and T3 as local recurrence based on the fact that pathological findings for T2 and T3 are consistent with those of T1. The fact that T1−T3 shared rare simultaneous mutations in *KRAS* and *SMAD4* support the idea that T2 and T3 recurred from T1. Nevertheless, the possibility that T2 and T3 are secondary cancers distinct from T1 cannot be ruled out completely because there is no evidence so far, on the difference in mutational signatures between locally recurrent tumors and secondary tumors. Fifth, we did not analyze samples at first recurrence because our aim was to identify mutational signatures associated with radioresistance by comparing mutational profiles between treatment-naïve tumors and tumors that recurred after radiotherapy; therefore, analyzing samples from the first recurrence, which is a tumor that recurs after chemotherapy, was beyond the scope of the present study. Nevertheless, we confirmed the presence of mutations commonly detected in T1−T3, i.e., *PIK3CA* (E545K), *KRAS* (G12D), and *SMAD4* (R361C and R361H), by Sanger sequencing of tumor samples from the first recurrence, indicating that this tumor also recurred from T1 and shares the trunk mutational characteristics with T2 and T3 (Supplementary Figure 3). Sixth, Sanger sequencing results for *PIK3CA* (c.1633G>A) and *TET1* (c.242G>A), in which mutations in T2 were not detected, should have been validated using other methods, including real-time polymerase chain reaction (PCR) and droplet digital PCR. However, we were not able to conduct these experiments due to a shortage of sample. Lastly, the T3 specimen was smaller than the other specimens, which might affect smaller coverage for T3 compared to that for the others.

In summary, we identified rare simultaneous mutations in *KRAS* and *SMAD4* in a series of tumors that survived multiple rounds of radiotherapy and validated the association between the mutation signature and radioresistance by conducting meta-analysis of published *in vitro* data and isogenic cell-based experiments. Based on this pilot study, we propose that our analytical pipeline would be useful for identification of clinically relevant mutation signatures of radioresistance, and that this approach warrants validation in larger cohorts. The predictive value of the *KRAS^mt^/SMAD4^mt^* signature for radioresistance should also be evaluated in the clinic.

## MATERIALS AND METHODS

### Ethics

This study was approved by the Institutional Review Boards of Gunma University Hospital (approved number: 1109). Written informed consent was obtained from the patient. The study was conducted in accordance with the ethical principles of the Declaration of Helsinki.

### Tissue sample collection

Tumor tissues were collected by punch biopsy. Normal tissues of the uterine cervix were collected by surgery. The presence of malignant cells was pathologically confirmed in all tumor specimens, and the absence of malignant cells was likewise confirmed in the normal tissue specimen.

### DNA preparation

DNA was extracted from the FFPE tissues using the QIAamp DNA FFPE Tissue kit (Qiagen). The TaqMan RNase P Detection Reagents kit (Thermo Fisher Scientific) was used to quantify purified DNA.

### Semiconductor-based next-generation sequencing

DNA (40 ng) was subjected to multiplex PCR amplification using an Ion AmpliSeq Comprehensive Cancer Panel that covers 95.4% of the exons of the 409 cancer-related genes. Library preparation and sequencing with the Ion Torrent sequencer were performed as previously described [39–41]. The templates were sequenced after emulsion PCR with 6–8 samples per Ion PI chip using the Ion PI HI-Q Chef kit (Thermo Fisher Scientific).

### Identification of somatic mutations and CNVs

Somatic mutations and CNVs were determined as previously described, with minor modifications [42]. Briefly, the sequenced data were aligned to the Genome Reference Consortium Human Build 37 (hg19), and reads were counted using Torrent Suite version 5.0 (Thermo Fisher Scientific). Single-nucleotide variants, insertions, and deletions were determined using the Ion Reporter software 5.0 tumor-normal workflow (Thermo Fisher Scientific), in which germline variants were subtracted from the tumor variants. The following criteria were used as cutoffs: total coverage >20, variant coverage >10, variant frequency >5%, and minor allele frequency <0.1%. All identified single-nucleotide variants, insertions, and deletions were visually inspected using the Integrative Genomics Viewer software to filter out possible strand-specific errors, such as a mutation detected only in either the forward or reverse DNA strand [43]. The dbSNP database was used to exclude SNPs from the called variants. SIFT, Polyphen-2, and Grantham scores were used to estimate evolutionary conservation and the effects of amino-acid substitution on the structure and function of the protein. CNVs were determined with the Ion Reporter software using an algorithm based on a Hidden Markov Model. To verify the robustness of next-generation sequencing, Sanger sequencing was performed for the somatic non-synonymous mutations in *PIK3CA*, *KRAS*, and *SMAD4* that were commonly identified in T1−T3, as well as for those in *KMT2A*, *TET1*, and *NLRP1* that were commonly identified in T2 and T3 but not in T1. As a result, the mutational status for 93.7% (30/32) of the samples analyzed was consistent with that observed after next-generation sequencing, demonstrating the robustness of the analytical workflow (Supplementary Figures 4 and 5).

### Identification of *KRAS*^mt^/*SMAD4*^mt^ and Control cell lines in CCLE

The *KRAS*^mt^/*SMAD4*^mt^ and Control cell lines were identified based on genetic information in cBioportal for Cancer Genomics (Table 2) [44, 45]. The *KRAS*^mt^/*SMAD4*^mt^ cell lines were derived from pancreatic, colorectal, stomach, and lung cancers. Control cell lines were selected randomly from CCLE, matching the cancer type to the *KRAS*^mt^/*SMAD4*^mt^ cell lines. Differences in the distribution of cancer types between the *KRAS*^mt^/*SMAD4*^mt^ and Control cell lines were not statistically significant (*P* = 0.68, as assessed by Fisher's exact test). The prevalence of *KRAS* and *SMAD4* mutations in clinical tumor specimens of pancreatic, colorectal, stomach, and lung cancer was confirmed using cBioportal for Cancer Genomics, and the combined prevalence of the mutations in the four cancer types was calculated by matching the relative proportion for each cancer type to those of the Control cell lines (Supplementary Table 3).

### Acquisition of SF_2_ data from the literature

All published SF_2_ data for the *KRAS*^mt^/*SMAD4*^mt^ and Control cell lines were obtained as previously described [20]. Briefly, a PubMed search was performed for each cell line using the terms “[cell line name] AND (X-rays OR gamma rays OR radiation)”. Two radiation oncologists (EN and TBMP) examined all manuscripts identified by the search in their entirety, and identified publications reporting data obtained from clonogenic assays after treatment with X-rays or γ-rays alone. This examination identified 95 relevant papers, from which SF_2_ data were obtained (Table 2 and Supplementary Table 4). For each cell line, the average SF_2_ was calculated and used for analysis.

### Cell culture

SW48 and SW48-*KRAS*^G12D^ cells were purchased from ATCC and cultured in DMEM (Gibco) supplemented with 10% fetal bovine serum (Sigma).

### siRNA knockdown

Transfection of siRNA was performed using HiPerFect (Qiagen), as previously described [46]. Briefly, siSMAD4 (Thermo Fisher Scientific, 4390824) or Control-siRNA (siControl, GGGAUACCUAGACGUUCUAdTdT; Sigma) was added to suspended cells after trypsinization. After 24 h, cells were trypsinized, suspended, and re-transfected with the siRNAs. Cells were incubated for 24 h after the second transfection and then subjected to clonogenic assay and immunoblotting (in parallel).

### Clonogenic assay

The clonogenic assay was performed as previously described [47]. Briefly, cells were seeded in 6 well plates at the specified numbers and then exposed to X-ray irradiation using a Faxitron RX-650 apparatus (100 kVp, 1.14 Gy/min; Faxitron Bioptics). After incubating for 10 days, cells were fixed with methanol and stained with crystal violet. Colonies comprising at least 50 cells were counted. The surviving fraction of irradiated cells was normalized to that of the corresponding unirradiated Controls.

### Immunoblotting

Immunoblotting was performed as previously described [48]. The following antibodies were purchased from Cell Signaling Technology: SMAD4 (38454) and β-actin (3700). Uncropped versions of the immunoblots are shown in Supplementary Figure 6.

### Statistical analysis

Differences in SF_2_ between two groups were examined as follows: First, normality was confirmed using the Shapiro-Wilk test. Next, variance was examined using an *F* test. Differences between two groups with equal variance were examined using Student's *t*-test. Differences between groups without equal variance were examined using Welch's *t*-test. Differences in the distribution of cancer type between the *KRAS*^mt^/*SMAD4*^mt^ cell lines and Control cell lines were examined using Fisher's exact test. All statistical analyses were performed using Prism 6 (GraphPad) or EZR (Saitama Medical Center), which is a graphical user interface for R ver. 3.3.2 (The R Foundation for Statistical Computing) [49]. A *P* value < 0.05 was considered significant.

## SUPPLEMENTARY MATERIALS FIGURES AND TABLES







## Abbreviations

CCLE: Cancer Cell Line Encyclopedia
CNVs: copy number variations
FFPE: formalin-fixed paraffin-embedded
PCR: polymerase chain reaction
SF_2_: the surviving fraction after 2-Gy irradiation as assessed by clonogenic assays
siControl: Control-siRNA
siSMAD4: SMAD4-siRNA.
