# Supplementary material for: Mutational analysis of uterine cervical cancer that survived multiple rounds of radiotherapy
Source: Oncotarget. 2018 Aug 24;9(66):32642–52. doi: 10.18632/oncotarget.25982 (PMC6135691; doi:10.18632/oncotarget.25982)
Supplement: Supplementary file 1 [file oncotarget-09-32642-s001.pdf]

Mutational analysis of uterine cervical cancer that survived multiple rounds of radiotherapy

SUPPLEMENTARY MATERIALS

ABL1	BCL9	CHEK1	EPHB4	FLT4	IKZF1	MAGEA1	MYB	PAX7	PTEN	SMARCA4	TOP1
ABL2	BCR	CHEK2	EPHB6	FN1	IL2	MAGI1	MYC	PAX8	PTGS2	SMARCB1	TP53
ACVR2A	BIRC2	CIC	ERBB2	FOXL2	IL21R	MALT1	MYCL1	PBRM1	PTPN11	SMO	TPR
ADAMTS20	BIRC3	CKS1B	ERBB3	FOXO1	IL6ST	MAML2	MYCN	PBX1	PTPRD	SMUG1	TRIM24
AFF1	BIRC5	CMPK1	ERBB4	FOXO3	IL7R	MAP2K1	MYD88	PDE4DIP	PTPRT	SOCS1	TRIM33
AFF3	BLM	COL1A1	ERCC1	FOXP1	ING4	MAP2K2	MYH11	PDGFB	RAD50	SOX11	TRIP11
AKAP9	BLNK	CRBN	ERCC2	FOXP4	IRF4	MAP2K4	MYH9	PDGFRA	RAF1	SOX2	TRRAP
AKT1	BMPR1A	CREB1	ERCC3	FZR1	IRS2	MAP3K7	NBN	PDGFRB	RALGDS	SRC	TSC1
AKT2	BRAF	CREBBP	ERCC4	G6PD	ITGA10	MAPK1	NCOA1	PER1	RARA	SSX1	TSC2
AKT3	BRD3	CRKL	ERCC5	GATA1	ITGA9	MAPK8	NCOA2	PGAP3	RB1	STK11	TSHR
ALK	BRIP1	CRTC1	ERG	GATA2	ITGB2	MARK1	NCOA4	PHOX2B	RECQL4	STK36	UBR5
APC	BTK	CSF1R	ESR1	GATA3	ITGB3	MARK4	NF1	PIK3C2B	REL	SUFU	UGT1A1
AR	BUB1B	CSMD3	ETS1	GDNF	JAK1	MBD1	NF2	PIK3CA	RET	SYK	USP9X
ARID1A	CARD11	CTNNA1	ETV1	GNA11	JAK2	MCL1	NFE2L2	PIK3CB	RHOH	SYNE1	VHL
ARID2	CASC5	CTNNB1	ETV4	GNAQ	JAK3	MDM2	NFKB1	PIK3CD	RNASEL	TAF1	WAS
ARNT	CBL	CYLD	EXT1	GNAS	JUN	MDM4	NFKB2	PIK3CG	RNF2	TAF1L	WHSC1
ASXL1	CCND1	CYP2C19	EXT2	GPR124	KAT6A	MEN1	NIN	PIK3R1	RNF213	TAL1	WRN
ATF1	CCND2	CYP2D6	EZH2	GRM8	KAT6B	MET	NKX2-1	PIK3R2	ROS1	TBX22	WT1
ATM	CCNE1	DAXX	FAM123B	GUCY1A2	KDM5C	MITF	NLRP1	PIM1	RPS6KA2	TCF12	XPA
ATR	CD79A	DCC	FANCA	HCAR1	KDM6A	MLH1	NOTCH1	PKHD1	RRM1	TCF3	XPC
ATRX	CD79B	DDB2	FANCC	HIF1A	KDR	MLL	NOTCH2	PLAG1	RUNX1	TCF7L1	XPO1
AURKA	CDC73	DDIT3	FANCD2	HLF	KEAP1	MLL2	NOTCH4	PLCG1	RUNX1T1	TCF7L2	XRCC2
AURKB	CDH1	DDR2	FANCF	HNF1A	KIT	MLL3	NPM1	PLEKHG5	SAMD9	TCL1A	ZNF384
AURKC	CDH11	DEK	FANCG	HOOK3	KLF6	MLLT10	NRAS	PML	SBDS	TET1	ZNF521
AXL	CDH2	DICER1	FAS	HRAS	KRAS	MMP2	NSD1	PMS1	SDHA	TET2	
BAI3	CDH20	DNMT3A	FBXW7	HSP90AA1	LAMP1	MN1	NTRK1	PMS2	SDHB	TFE3	
BAP1	CDH5	DPYD	FGFR1	HSP90AB1	LCK	MPL	NTRK3	POT1	SDHC	TGFBR2	
BCL10	CDK12	DST	FGFR2	ICK	LIFR	MRE11A	NUMA1	POU5F1	SDHD	TGM7	
BCL11A	CDK4	EGFR	FGFR3	IDH1	LPHN3	MSH2	NUP214	PPARG	SEPT9	THBS1	
BCL11B	CDK6	EML4	FGFR4	IDH2	LPP	MSH6	NUP98	PPP2R1A	SETD2	TIMP3	
BCL2	CDK8	EP300	FH	IGF1R	LRP1B	MTOR	PAK3	PRDM1	SF3B1	TLR4	
BCL2L1	CDKN2A	EP400	FLCN	IGF2	LTF	MTR	PALB2	PRKAR1A	SGK1	TLX1	
BCL2L2	CDKN2B	EPHA3	FLI1	IGF2R	LTK	MTRR	PARP1	PRKDC	SH2D1A	TNFAIP3	
BCL3	CDKN2C	EPHA7	FLT1	IKBKB	MAF	MUC1	PAX3	PSIP1	SMAD2	TNFRSF14	
BCL6	CEBPA	EPHB1	FLT3	IKBKE	MAFB	MUTYH	PAX5	PTCH1	SMAD4	TNK2	

Supplementary Figure 1: Ion ampliseq comprehensive cancer panel gene list.

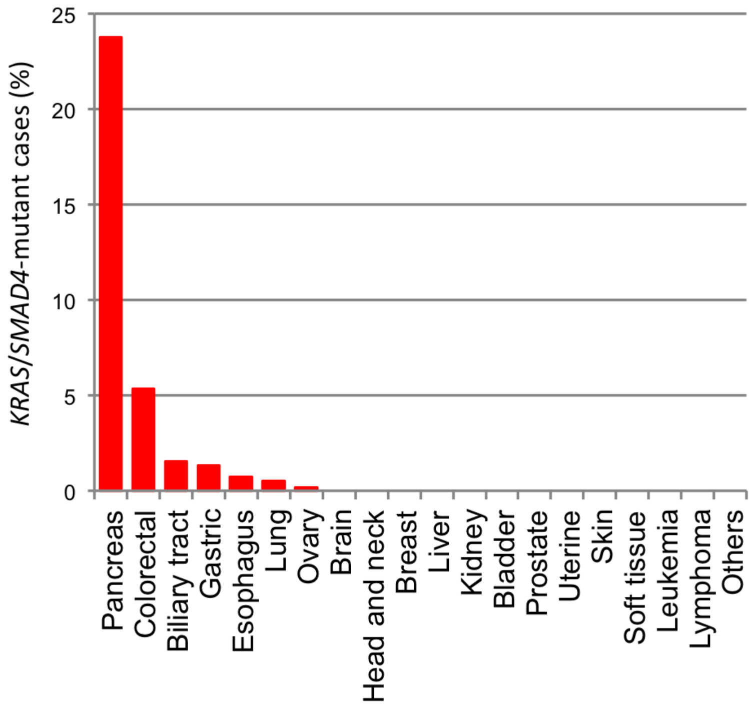

Supplementary Figure 2: Frequency for simultaneous alterations in *KRAS* and *SMAD4* according to tumor type. *n* = 22928. The analysis was performed using cBioportal [45]. See Supplementary Table 5 for the data set used.

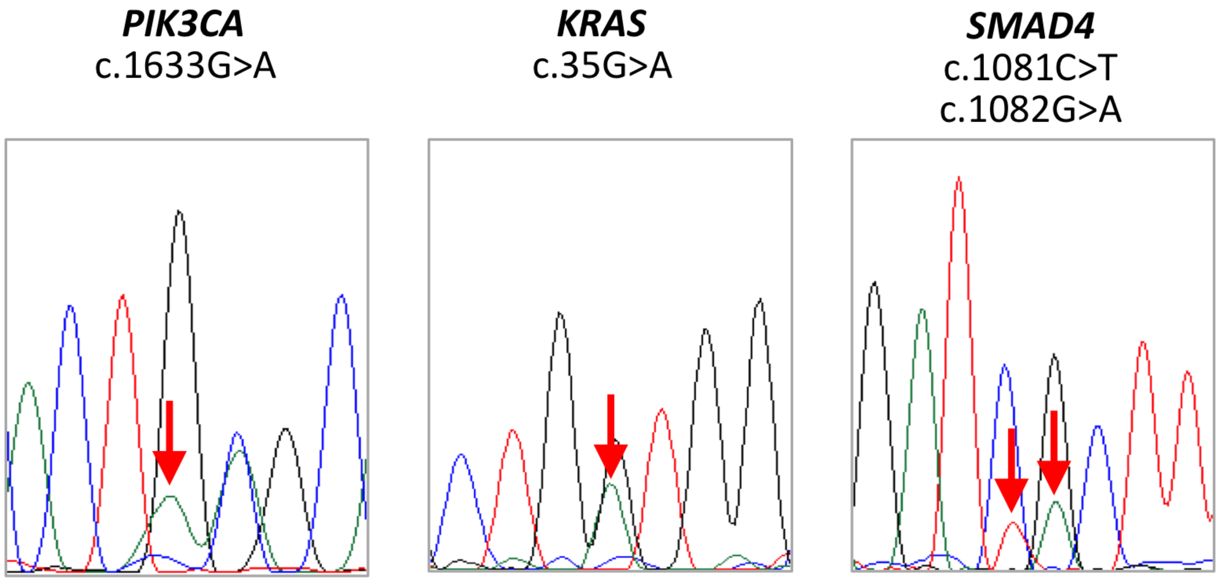

Supplementary Figure 3: Sanger sequencing results for the mutations in *PIK3CA* (c.1633G>A), *KRAS* (c.35G>A), and *SMAD4* (c.1081C>T and c.1082G>A) in the tumor sample of the first recurrence. Red arrows show variant.

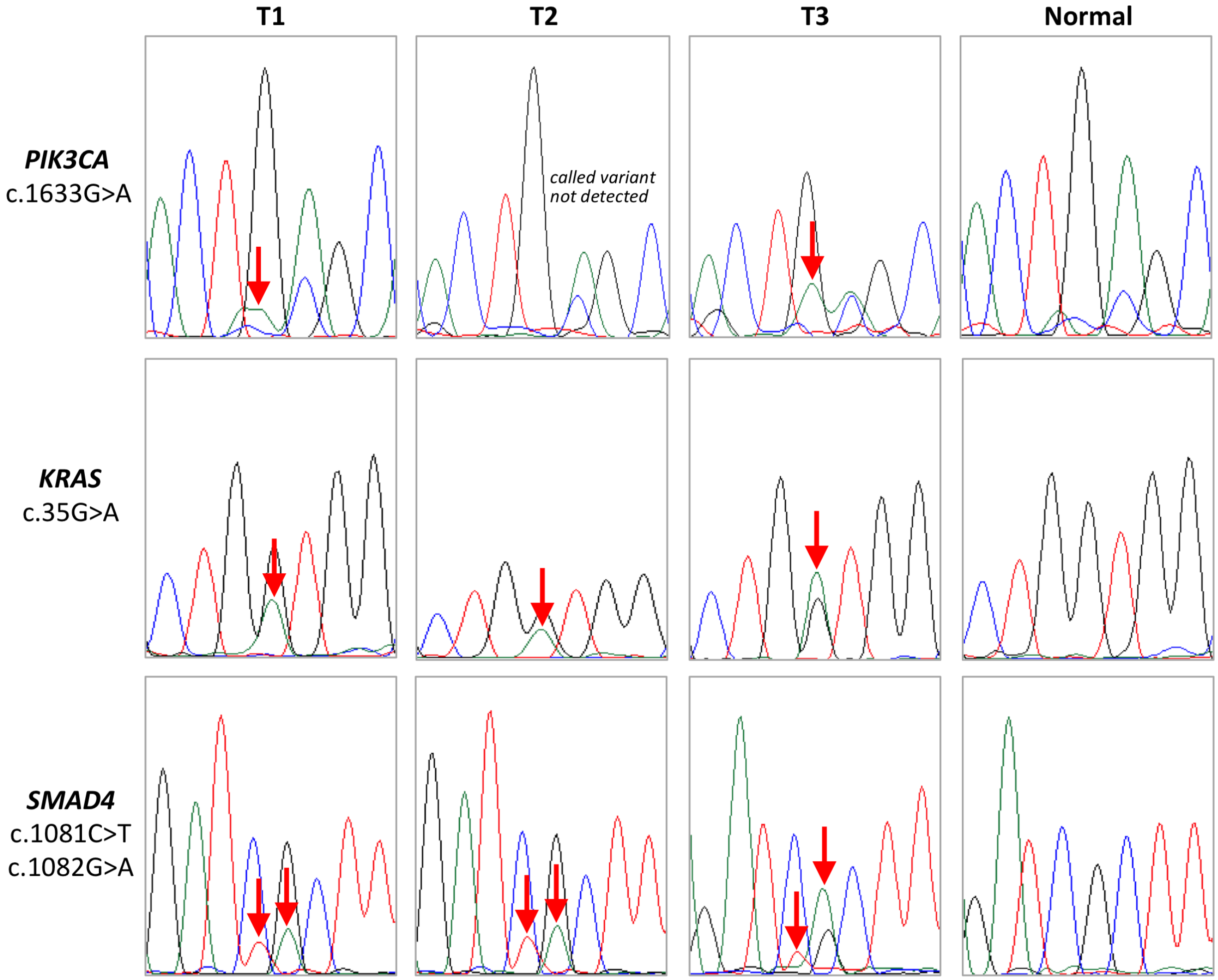

Supplementary Figure 4: Verification of identified mutations in *PIK3CA*, *KRAS* and *SMAD4* by Sanger sequencing. Sanger sequencing traces for the genes commonly identified in T1, T2 and T3 are shown. Red arrows show variant. For *PIK3CA*, it is considered that the base next to the somatic mutation (c.1633G>A) in the normal sample is a SNP (A/C).

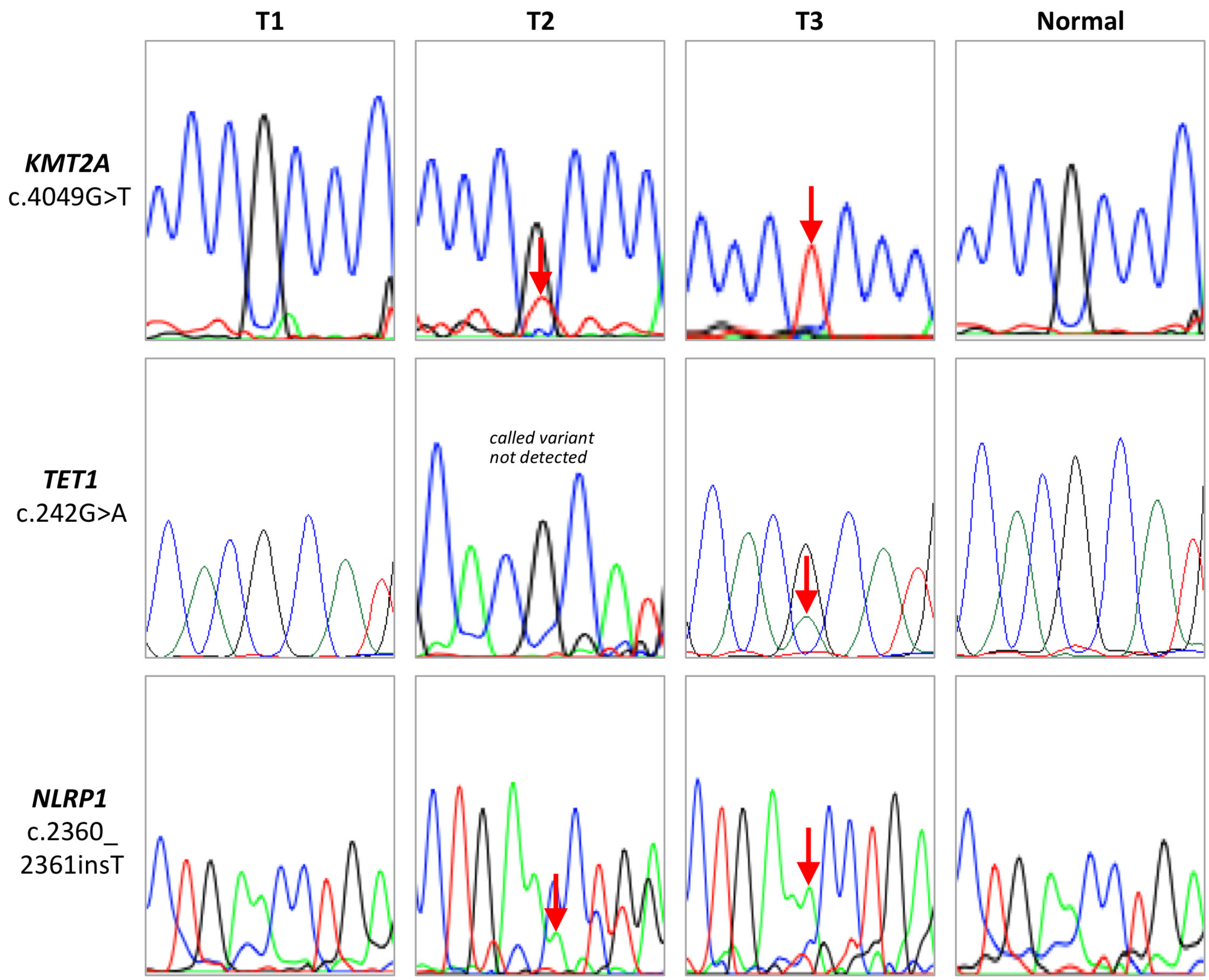

Supplementary Figure 5: Verification of identified mutations in *KMT2A*, *TET1* and *NLRP1* by Sanger sequencing. Sanger sequencing traces for the genes commonly identified in T2 and T3 but not in T1 are shown. Red arrows show variant.

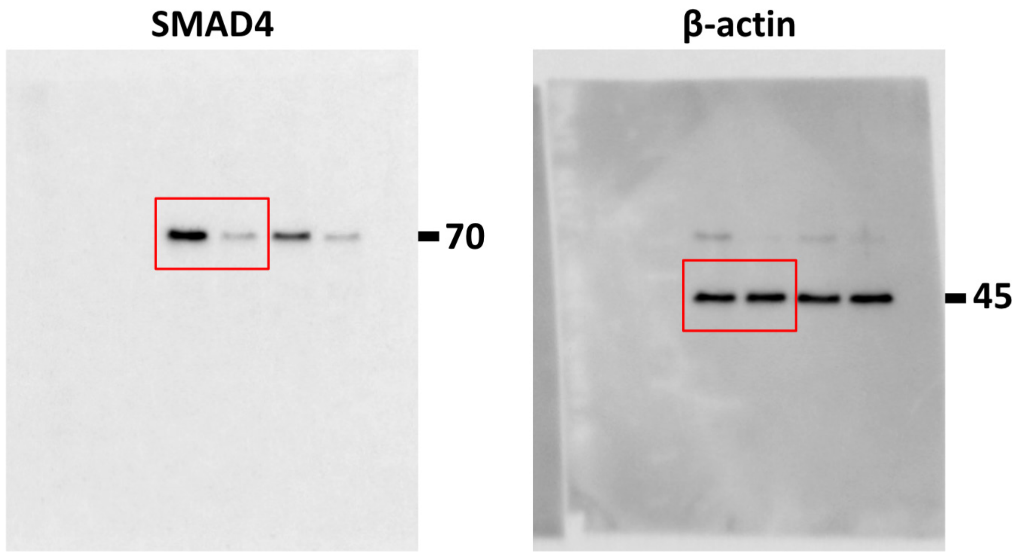

Supplementary Figure 6: Uncropped immunoblots presented in Figure 3C.

Supplementary Table 1: Overview of targeted amplicon sequencing results

Sample	Mapped reads	Reads on target (%)	Coverage depth	Uniformity (%)	Coverage (%)				#of somatic mutation			#of CNV
					1×	20×	100×	500×	Nonsynon	Synon	Total	
T1	10475349	99.11	657.5	91.51	99.64	98.02	93.43	58.70	5	0	6	17
T2	10899935	99.31	686.6	88.46	99.48	97.05	91.19	57.23	11	1	12	32
T3	8804867	80.12	352.8	59.06	89.96	84.98	60.14	15.91	75	6	81	345
Normal	12331540	98.28	770.2	95.30	99.82	98.82	96.70	75.49	NA	NA	NA	NA

Abbreviations: Nonsynon; non-synonymous, synon; synonymous, CNV; copy number variation; NA; not assessible.

Supplementary Table 2: Somatic non-synonymous mutations identified in this study

Gene	T1	T2	T3
AKAP9			p.Glu1585Ala
AKT3			p.Met460Arg
ALK			p.Trp593Cys
AMER1			p.Arg871Gln
ARID1A			p.Ser2142Arg
ARID2			p.Pro1019Arg
ARNT			p.Ser539Thr
ATF1			p.Gln153Arg
AXL			p.Tyr498Cys
BAI3	p.Cys1269Ter		
BCL11B			p.Pro51His
CD79B			p.Glu25Val; p.Thr139Ala
CDK12			p.Glu1193Gly
CIC		p.Gly1287Arg	
CREBBP			p.Leu1098Pro
CRTC1		p.Gly501Arg	
CSF1R			p.Ala505Asp
CTNNB1			p.Tyr724Cys
ERCC5			p.Asp77Ala
ETV4			p.Ile95Ser
FANCG			p.Ser344Arg
FBXW7			p.Asn621Ser
FLI1			p.Val11Leu
FLT4			p.Pro138Thr
FOXP4		p.Ala610Val	
HCAR1			p.Ala60Ser
IGF2R			p.Leu2008Val
KAT6A			p.Gln1283Arg; p.Ser1576Cys
KDM5C			p.Cys1202Arg
KMT2A		p.Arg1350Leu	p.Arg1350Leu
KMT2C			p.Ala2223Glu; p.Arg4828Cys
KMT2D			p.Ser901Phe; p.Pro916Leu; p.Gly1946Val, COSM548519; p.Glu3216Ter, COSM221123
KRAS	p.Gly12Asp, COSM1135366	p.Gly12Asp, COSM1135366	p.Gly12Asp, COSM1135366

LCK			p.Leu342Gln
MAML2			p.Ala521Asp
MYH11			p.Ala1396Asp
NCOA1			p.Arg1207Gly
NIN			p.Ser144Asn
NLRP1		p.Arg788fs	p.Arg788fs
NOTCH1			p.Asp2082Glu
NOTCH4		p.Arg1793Gln; p.Arg130Lys	
NUP98			p.Ala1602Asp
PAX3			p.Met299Lys; p.Ser497Arg
PAX7			p.Ser439Phe
PDE4DIP			p.Lys1104Asn
PER1			p.Ala1254Asp
PIK3CA	p.Glu545Lys, COSM763	p.Glu545Lys, COSM763	p.Glu545Lys, COSM763
PMS2			p.Gln449Leu, COSM747348
PTCH1			p.Ser640Arg
RAF1			p.Ala314Val
RALGDS			p.Ala763Asp
RNF213			p.Gly1828Ala; p.Ser3939Gly; p.His4391Gln
ROS1			p.Val213Gly
RUNX1T1			p.Gly130Ala
SAMD9			p.Asn1141fs, COSM602765
SDHD			p.Ala9Gly
SETD2			p.Gly1563Ala; p.Ser1992Arg
SMAD4	p.Arg361Cys, COSM14140; p.Arg361His, COSM14122	p.Arg361Cys, COSM14140; p.Arg361His, COSM14122	p.Arg361Cys, COSM14140; p.Arg361His, COSM14122
STK36			p.Gln1074Ter
SYNE1			p.Val3180Gly; p.Asn6582Lys; p.Cys7960Ser
TAF1L			p.His1077Arg
TCF7L1			p.Gln433Ter
TET1		p.Arg81His, COSM919743	p.Arg81His, COSM919743
TSC1			p.Pro391Thr
TSC2			p.Ala623Ser
USP9X			p.Tyr2220Cys
XPC			p.Cys771Ser
ZNF384			p.Asn407Lys

Supplementary Table 3: Prevalence of KRAS and SMAD4 mutations in pancreatic, colorectal, gastric and lung cancers in clinical tumors

Cancer type	Control cell lines		Clinical tumors					
	# of cell lines	Sample size (%)	# of case			Sample size-matched prevalence (%)		Data source
			KRAS^{mt} only	*SMAD4*^{mt} only	Total	*KRAS*^{mt} only	*SMAD4*^{mt} only	
Pancreatic	3	25	310	1	383	20	0	QCMG, Nature 2016
Colorectal	5	41	96	4	212	19	0.8	TCGA, Nature 2012
Gastric	2	17	79	21	287	4.6	1.2	TCG, Nature 2014
Lung	2	17	300	31	1144	4.4	0	TCGA, Nat Genet 2016
Total	12	100	785	57	2026	**48**	**2.5**	-

Supplementary Table 4: Publications containing SF_2 data for *KRAS*^{mt}/*SMAD4*^{mt} and Control cell lines. See Supplementary_Table_4

Supplementary Table 5: Data set used for frequency analysis on simultaneous *KRAS*/*SMAD4* alterations in various types of tumors. See Supplementary_Table_5

Supplementary Table 6: *P* values for statistical analyses for differences in SF_2

Test	Data for figure 3A	Data for figure 3B
Shapiro–Wilk test	0.083	0.084
F test	0.039	0.076
Student's *t*-test	NA	0.034
Welch's *t*-test	0.010	NA

Abbreviations: NA, not assessed.
